# Bioinformatics Study of Structural Patterns in Plant MicroRNA Precursors

**DOI:** 10.1155/2017/6783010

**Published:** 2017-02-09

**Authors:** J. Miskiewicz, K. Tomczyk, A. Mickiewicz, J. Sarzynska, M. Szachniuk

**Affiliations:** ^1^Institute of Computing Science and European Centre for Bioinformatics and Genomics, Poznan University of Technology, Poznan, Poland; ^2^Institute of Bioorganic Chemistry, Polish Academy of Sciences, Poznan, Poland

## Abstract

According to the RNA world theory, RNAs which stored genetic information and catalyzed chemical reactions had their contribution in the formation of current living organisms. In recent years, researchers studied this molecule diversity, i.a. focusing on small non-coding regulatory RNAs. Among them, of particular interest is evolutionarily ancient, 19–24 nt molecule of microRNA (miRNA). It has been already recognized as a regulator of gene expression in eukaryotes. In plants, miRNA plays a key role in the response to stress conditions and it participates in the process of growth and development. MicroRNAs originate from primary transcripts (pri-miRNA) encoded in the nuclear genome. They are processed from single-stranded stem-loop RNA precursors containing hairpin structures. While the mechanism of mature miRNA production in animals is better understood, its biogenesis in plants remains less clear. Herein, we present the results of bioinformatics analysis aimed at discovering how plant microRNAs are recognized within their precursors (pre-miRNAs). The study has been focused on sequential and structural motif identification in the neighbourhood of microRNA.

## 1. Introduction

For many years, computational methods have been applied to support wet-lab experiment in biologically oriented study.* In silico* methods can help in discriminating ineffective experimental approaches or indicate the most promising ones. On the other hand,* in vivo* and* in vitro* experiments enable validation of hypotheses proposed in computational phase and may confirm or contradict them.

Solving biological problems with the use of computational methods appeared successful in various domains [1–11]. In our work, we consider an issue of microRNA recognition and we apply bioinformatics methods aiming to explain how this molecule is formed within living organisms of the plant kingdom.

MicroRNAs constitute a group of small, non-coding single-stranded RNAs of ~22 nt in length, involved in post-transcriptional regulation of gene expression [6, 12–15]. These molecules are widespread in genomes of animals and plants [6, 16]. Their biogenesis is a complex process which differs between organisms. The biogenesis of miRNA starts in the nucleus, with transcription process of primary miRNA (pri-miRNA) performed by RNA polymerase II [17, 18]. The transcript forms long hairpin loop structure which consists of single- and double-stranded regions. In double-stranded regions, single mismatches (i.e., noncomplementary nucleotides), internal loops, and bulges can be found in numerous locations [19, 20]. In animals, pri-miRNA is further processed by Drosha enzyme (ribonuclease III endonuclease). In collaboration with the RNA-binding protein, Drosha cleaves primary transcript of miRNA to the ~70 nt long precursor (pre-miRNA) [17, 21, 22]. For animals, this is the last step of the nuclear stage. Created stem-loop structure is transferred to the cytoplasm where another enzyme, called Dicer, cleaves miRNA:miRNA^*∗*^ duplex out of pre-miRNA [22–24]. Ribonuclease Dicer acts as a molecular ruler: it counts the distance between 3′ or 5′ end and the cleavage site; next, it performs the cut which releases the miRNA:miRNA^*∗*^ duplex [25–27]. In plants, the maturation process of miRNA is slightly different. After creation of plant pri-miRNA, a complex consisting of Dicer Like 1 enzyme (DCL1), the double-stranded RNA-binding protein HYL1 (hyponastic leaves 1), and the zinc-finger protein SE (serrate), with an assistance of the nuclear cap-binding complex, cleaves pri-miRNA to pre-miRNA [22, 28]. Before being transported to the cytoplasm, DCL1 performs at least two cleavages to release miRNA:miRNA^*∗*^ duplex from the precursor structure. There are two miRNA cleavage mechanisms in plants. In base-to-loop cleavage mechanism, the first cut is done in the lower stem region and the second cut in the upper (loop) region. In loop-to-base mechanism, DCL1 cuts in the opposite direction. After being cleaved, the duplex is transferred outside the nucleus. This double-stranded miRNA:miRNA^*∗*^ duplex is the result of a primary transcript maturation process. miRNA constitutes one strand and miRNA^*∗*^ is located on the complementary one. These two strands are next separated by Argonaute (AGO) protein, the main part of RNA-induced silencing complex (RISC) [17, 23, 28]. In most cases, miRNA^*∗*^ is degraded after its separation from miRNA [17, 28]. Thus, activated molecular version of miRNA molecule is a single-stranded RNA. After creation of single-stranded mature miRNA and embedding it to the complex (given a mi-RISC complex in result), miRNA guides this complex to mRNA with the complementary sequence. mi-RISC enables degradation of the target mRNA or inhibition of the translation process [29–32].

Compared to the biogenesis of miRNA in animals, which is better understood, maturation of plant miRNA still has some unresolved issues. One of them is recognition of the miRNA:miRNA^*∗*^ duplex in miRNA precursor by a microprocessor complex, containing DCL1, HYL1, and SE proteins. This microprocessor complex performs at least two cuts in order to release the miRNA:miRNA^*∗*^ duplex (miRNA on one strand and complementary sequence of miRNA^*∗*^ on the other strand) from pre-miRNA molecule. Yet, it has not been discovered how this duplex is recognized within the precursor structure. It is supposed that some structural motifs, appearing in the vicinity of microRNA [20, 33–35], guide the DCL1 enzyme where to perform the cutting. The importance of miRNA neighbouring regions where the DCL1 enzyme starts cleaving has been already experimentally confirmed on the secondary structure level [20, 33–38]. Thus, we assume that irregulations in primary and secondary structures may be the signal for DCL1 where to starts cleaving.

Recent research concerning miRNAs has suggested the role of proteins in microprocessor complex (HYL1 and SE) in miRNA recognition. The proper selection of cutting sites is poorly understood in both pri-miRNA and pre-miRNA, but most probably it depends on HYL1 [39–41]. The importance of mismatches occurring in double-stranded regions of miRNA:miRNA^*∗*^ duplex was also revealed. Mismatches can influence the length of the mature microRNA, producing either longer [42–44] or shorter molecules [19]. It has been proven that miRNA genes can contain introns which are strictly correlated with biogenesis and proper functioning of their host miRNAs [12]. However, in spite of all this information about plant miRNAs, we still do not know how microprocessor complex enzyme recognizes the miRNA:miRNA^*∗*^ duplex within its precursor.

The presented analysis, aimed at helping in answering this issue, was performed according to the following steps: (i) creating a set of plant miRNA sequences with experimentally confirmed cleavage mechanisms, (ii) downloading precursor sequences of selected miRNAs and supplementing them to the desired length, (iii) analysing sequences using WebLOGO [45] and MEME Suite [46], (iv) predicting secondary structures of miRNA precursors by RNAstructure [4] and mfold [9], (v) choosing the best secondary structures for further analysis, and (vi) analysing predicted secondary structures in the search for structural patterns. In the paper, we present consecutive analytical steps, starting from Section 2. In Section 3, we present the results of our work. Finally, the discussion of results and future plans are presented.

## 2. Materials and Methods

In our research, we have decided to analyse microRNA precursors in plants on two structural levels, the sequence and the secondary structure, using selected bioinformatic tools. The sequences of pre-miRNAs were derived from publicly available data sources. First, 50 plant miRNAs with experimentally confirmed cleavage mechanism (base-to-loop or loop-to-base) were selected based on [37]. This preliminary dataset *S*1 consisted of

- 38 miRNAs with base-to-loop mechanism: mir164b, mir165a, mir165b, mir167a, mir167b, mir167d, mir168a, mir168b, mir169a, mir170, mir171a, mir172a, mir172b, mir172d, mir172e, mir390a, mir390b, mir391, mir393a, mir393b, mir395a, mir395b, mir395c, mir396b, mir397a, mir398b, mir398c, mir399b, mir399c, mir408, mir827, ymir158a, ymir403, ymir771, ymir824, ymir864, ymir161, ymir400, ymir825, and mir164c,
- 12 miRNAs with loop-to-base mechanism: ymir400, ymir825, mir156a, mir156b, mir156c, mir156d, mir156h, mir160a, mir160b, mir160c, mir171b, and mir171c.

**Figure 1:**
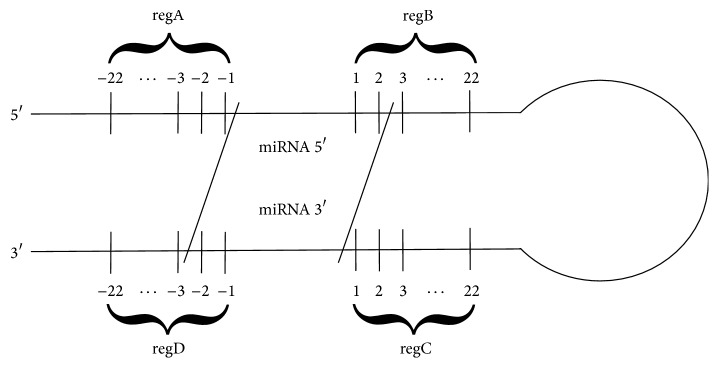
Schematic view of pre-miRNA with annotated miRNA:miRNA^*∗*^ vicinity regions.* regA* and* regD* are located in the lower stem and* regB* and* regC* in the upper stem.

Next, precursors including sequences from the preliminary set *S*1 were searched in miRBase [47]. Sequences of these pre-miRNAs were downloaded for further analysis and collected in set *S*2. Taking into account previous results [20, 33–35], we have assumed that a region recognized by the microprocessor complex is located in the closest vicinity of the miRNA:miRNA^*∗*^ duplex. Thus, we planned to analyse ca 22 nt-long fragments neighbouring miRNA from both the base and the loop side, and we needed all pre-miRNA sequences in *S*2 to have at least 22 nucleotides in every strand of the region between miRNA:miRNA^*∗*^ and 3′/5′ ends. Some sequences that did not satisfy this condition were supplemented based on [37] and* Arabidopsis thaliana* genome stored completely in the TAIR database [48]. After collecting in *S*2 sequences of the required length, miRNA vicinity was annotated in each instance. In lower stem, we distinguished* regA* region in 5′ strand and* regD* in the opposite strand. Upper stem included* regB* region in 5′ strand and* regC* in 3′ strand (Figure 1).

The first analytical step was done using WebLOGO [45] and MEME Suite [46]. WebLOGO tool finds the relative frequency of particular nucleotide type at each position of the sequence within multiple sequence alignment. MEME Suite is a motif-based sequence analysis tool. MEME was run with default parameter values, except for minimum width set to 3 and maximum width set to 4. Thus, MEME was tuned to look for 3-4-nucleotide-long sequence motifs [49, 50]. Next, the secondary structures of pre-miRNAs from *S*2 were predicted using RNAstructure [4] and mfold [9]. For both programs default parameter values were applied, except for the temperature in RNAstructure that was set to 295.15 K (22°C). Finally, the secondary structures were processed using own script that searched for predefined structural patterns, like bulges and symmetric and asymmetric loops. Structural pattern (motif) of our interest was defined as double-stranded structure fragment closed by canonical base-pairs on both ends, having up to 10 nucleotides in each strand and including at least one unpaired nucleotide (in any strand).

**Figure 2:**
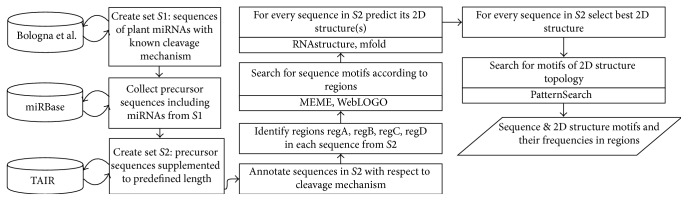
Consecutive steps of bioinformatics analysis.

Figure 2 presents the main steps of our bioinformatic analysis.

## 3. Results

In the preparation step preceding an analysis, we have collected basic information about primary structures of plant miRNAs. Next, set *S*2 was created and subjected to processing by bioinformatics tools. The set contained precursor sequences derived from* Arabidopsis thaliana* for which cleavage sites and cleavage mechanisms were identified and confirmed experimentally [37]. As a reference, we prepared a set of 50 random sequences, each of 22-nucleotide length, which correspond to miRNA:miRNA^*∗*^ vicinity regions.

The analysis started from running MEME Suite aimed at searching for sequence patterns. Sequences were encoded using 4-letter alphabet A,C,G,U representing four nucleotides building up RNA molecule: Adenine, Cytosine, Guanine, and Uracil, respectively. It has been decided to look for 3-4-nucleotide-long sequence motifs located between the 1st and the 22nd nucleotide beyond the miRNA:miRNA^*∗*^ duplex. Motifs were searched in the first-cut regions, that is,* regA* and* regD* in sequences with base-to-loop mechanism and* regC* and* regB* in loop-to-base sequences. We combined these regions based on the cleavage sites and 5′-3′ strand orientation. The numbering of nucleotides in miRNA vicinity goes from −1 to −22, where nucleotide −1 is the first nucleotide beyond miRNA. The number of each motif at particular position of the sequence is shown in Figure S2 (in Supplementary Material available online at https://doi.org/10.1155/2017/6783010).

MEME results obtained for the sequences including first-cut regions are displayed in Table 1. It appears that no motif occurs in more than 25% of these sequences, but a comparison to random sequences shows significant differences between the sets.

**Figure 3:**
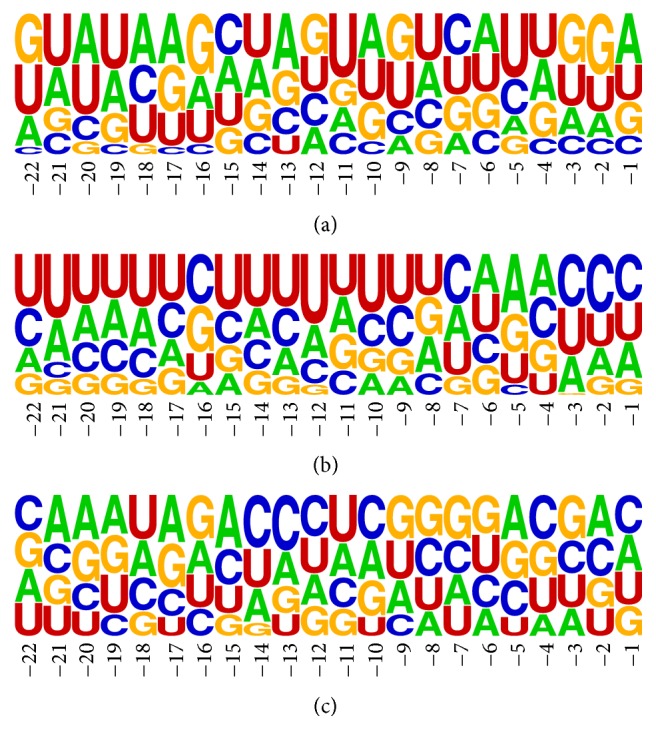
WebLOGO plots of nucleotide frequency in miRNA:miRNA^*∗*^ vicinity. First-cut region: (a)* regAC*, (b)* regBD*, and (c) random sequences.

The next analytical step concerned again regA and regC (further treated as single set and denoted as regAC), regB and regD (further denoted as regBD), and random sequences. We used WebLOGO tool to receive the information about nucleotide frequencies at particular position in miRNA vicinity. Nucleotides represented by respective letters positioned on top are the most frequent on associated positions (Figure 3 for first-cut regions, Figure S1 (Supplementary Material) for the second-cut regions). The general information concerning particular nucleotide occurrence in entire region(s) is provided in Table 2. From diagram *a* in Figure 3, showing the results for* regAC*, we can observe that U and C dominate at position −5. In the analogical region, for second cutting, the same position is occupied by A and U. Diagram* b* in Figure 3 with the results for* regBD* shows many pyrimidines (U or C). Particularly, C and U dominate positions 1–3 of the 3′ overhang. Purines (A and G) are overrepresented only at position 5. Despite these observations, WebLOGO results for* regAC* and* regBD* seemed similar to the results obtained for random sequences. Thus, two Student's *t*-tests of paired-samples were applied for

- each nucleotide occurrence (percentage values) at positions −22 to −1 in* regAC* and in random sequences,
- each nucleotide occurrence (percentage values) at positions −22 to −1 in* regBD* and in random sequences.

The resulting *p* values obtained for both tests were equal to 1.00. This revealed no significant difference between values for* regAC*/*regBD* and values for the random set. Percentages of each nucleotide occurrence in the first-cut regions provided by WebLOGO are displayed in Tables S1–S3 (Supplementary Material).

In the next step, the secondary structures were predicted from sequences of 50 pre-miRNAs from set *S*2. Every sequence was processed by RNAstructure and mfold which generated several output structures. The most compact structure was selected for every input sequence and passed for further analysis. In the majority of cases, the most compact was the structure displaying the minimum free energy.

Consequently, bulges and internal loops were searched and subjected to an analysis by self-developed script* PatternSearch*. Bulge is a structural motif formed in a double-stranded fragment where at least one nucleotide of one strand is unpaired. Internal loop has unpaired nucleotides in both strands. If the number of unpaired nucleotides is equal for both strands, the motif is known as symmetric internal loop. Otherwise, asymmetric internal loop is formed [51, 52]. In the manuscript, we use the following notation to encode secondary structure motif. Each motif is described by a pair of numbers U-W, which specify how many unpaired nucleotides are found in each strand of the double-stranded fragment. If U is equal to 0 (no unpaired nucleotide in one of the strands) and W is between 1 and 10, the corresponding motif is a W-nucleotide bulge. If both U and W are greater than 0, the corresponding motif is an internal loop. For example, 2-3 loop describes a motif composed of two strands, where there are 2 unpaired nucleotides in one of the strands (either 5′-3′ or 3′-5′) and 3 unpaired nucleotides in the other strand.

In this paper, we focused on regions where DCL1 performs the first cutting within the precursor structure, that is, lower stem (*regAD*) in structures with base-to-loop mechanism and upper stem (*regBC*) in case of loop-to-base mechanism. With regard to these vicinity regions, we have searched 50 secondary structures of pre-miRNAs for an occurrence of bulges and internal loops with different sizes (up to 10 nt on one of the strands).  Table 3 presents most numerous motifs found by the script. The numbers of particular secondary structure motifs (bulges and internal loops), with respect to the first-cut and the second-cut region, are presented in Figure S3 (Supplementary Material). The other motifs' total occurrence has not exceeded 10 in the whole dataset of secondary structures; thus, they were considered irrelevant.

An analysis of* PatternSearch* output suggested that an arrangement of bulges (0-1, 0-2, and 0-3 in Table 3) within pre-miRNA was random. Our study also shows that significantly more symmetric internal loops occur in the first-cut regions than in the region of the second cutting. Symmetric internal 1-1 loops (one unpaired nucleotide in each strand of the loop) appeared 3 times more often than bulges. In contrast to bulge arrangement, small internal loops demonstrated the tendency to locate in specific regions of the structure. In 90% of the analysed structures, we found symmetric internal 1-1 and 2-2 loops in the closest vicinity of miRNA:miRNA^*∗*^ duplex, that is, 1-5 nucleotides beyond the duplex (c.f.  Table 4).

**Figure 4:**
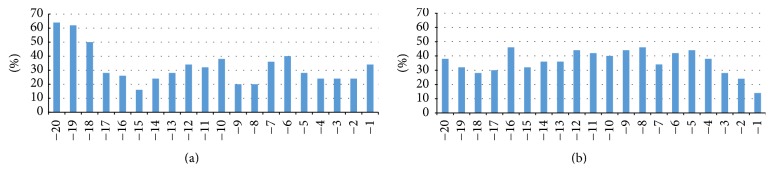
Percentage of unpaired nucleotides at specified positions in the first-cut regions: (a)* regAC* and (b)* regBD*.

We investigated the number of unpaired regions in* regAC* and* regBD* from first-cut mechanism (Figure 4). Thus, in the case of* regAC* analysis, we used* regA* of base-to-loop structures and* regC* of loop-to-base structures. In the case of* regBD*, we took* regB* of loop-to-base structures and* regD* of base-to-loop structures. In* regAC,* the most paired are positions −8, −9, and −15 (80%). On the three farthest positions mismatches are most frequently occurring (60%). On the other positions, the frequency of mismatches is approximately 30%. In contrast to* regAC*, where either regions with high paring or high mismatch level appear, in* regBD* the frequency of mismatches is very similar at each position.

An occurrence of small symmetric loops complies with the small number of mismatches distorting the stem in the region of the first cut performed by DCL1. This indicates potential structural pattern recognized by this enzyme. Occurrence of unpaired residues located further than 5 nt beyond miRNA:miRNA^*∗*^ duplex can also indicate potential position for the first cut.

## 4. Conclusions

In the paper, we focused on discovering motifs in primary and secondary structures of selected plant pre-microRNAs in order to answer the question how microprocessor composed of DCL1, HYL1, and SE recognizes the borders of microRNA:microRNA^*∗*^ duplex. The set of 50 sequences with experimentally confirmed cleavage mechanism was tested by selected bioinformatic tools. Sequence analysis was done using MEME Suite and WebLOGO tool. The results from MEME suggest that potential sequence motifs are UCUC in* regAC* and AACA, GUGG, and ACGG in* regBD*. This indicates that the sequence motifs could consist of either pyrimidines only (in* regAC*) or three purines and only one pyrimidine (in* regBD*). The results from WebLOGO tool were considered nonsignificant. An analysis of the secondary structure shows that the region in the vicinity of the first cut forms well defined stem comparing to the region of the second cut. However, it has been found that small symmetric internal loops 1-1 and 2-2 appear in up to 5 nt distance from the duplex. This constitutes a derogation from the results obtained for the experimentally solved RNA structures where 0-1 bulges are more common than internal loops 1-1 [53, 54]. These defined sequence and secondary structure patterns can play a key role in recognizing the location of miRNA:miRNA^*∗*^ duplex by DCL1 enzyme. To verify this theory, biochemical experiments involving artificially designed pre-miRNA [11] should be performed, which is planned to be done in the nearest future. Moreover, our future plans include prediction of the 3D structures of pre-miRNAs and their analysis with respect to characteristic structural features. For this purpose, computational tools like RNAComposer [3], MCQ4Structres [8], and PyMOL [55] will be applied. The generated 3D models will be evaluated based on their adjustment to the model of DCL1 structure. Finally, the analysis concerning three structural levels is going to be extended for all sequences of plant pre-miRNAs deposited in publicly available databases. However, it should be mentioned that for the majority of these sequences the cleavage mechanism has not been recognized yet.

## Supplementary Material

Supplementary Material include information about WebLOGO plot of nucleotide frequencies for second-cut regions (Fig S1), number of particular sequence motifs in the first-cut regions (Fig S2), percentage of each nucleotide on specific position in the vicinity of miRNA:miRNA∗ duplex for first-cut regions (Tab S1 & S2) and for random sequences (Tab S3), and number of particular secondary structure motifs in first- and second-cut regions (Fig S3).

## Figures and Tables

**Table 1:** MEME results for *regAC*, *regBD*, and random sequences.

Region	Sequence motif	Occurrence
regAC	UCUC	11 (22%)

regBD	AACA	9 (18%)
	GUGG	6 (12%)
	ACGG	5 (10%)

Random	GUGU, GUUC, GUUU…	2 (4%)

**Table 2:** Percentage of each nucleotide occurrence in *regAC*, *regBD*, and random sequences.

	First-cut region		Second-cut region		
	regAC	regBD	regAC	regBD	Random
A	26%	24%	30%	29%	26%
C	17%	26%	14%	21%	26%
G	26%	18%	21%	17%	25%
U	31%	33%	35%	34%	23%

**Table 3:** Number of relevant secondary structure motifs found in miRNA vicinity.

	Secondary structure motif								
	1-1	2-2	3-3	1-2	1-3	2-3	0-1	0-2	0-3
Base-to-loop									
Lower stem	42	12	8	13	3	8	16	6	3
Upper stem	26	9	5	7	10	2	9	4	7
Loop-to-base									
Lower stem	10	1	1	5	2	4	3	0	0
Upper stem	14	3	2	2	1	1	2	1	4

**Table 4:** Small internal loops found in close miRNA:miRNA^*∗*^ vicinity. The table shows the number and percentage of structures that have 1-1 and 2-2 loops at specified positions in miRNA:miRNA^*∗*^ vicinity.

Secondary structure motif	1-1 loop					2-2 loop		
Distance from miRNA [nt]	1	2	3	4	5	1	2	3
Number and percentage of structures with motif	19 38%	5 10%	6 12%	4 8%	2 4%	4 8%	2 4%	3 6%
