# Supplementary material for: Bioinformatics Study of Structural Patterns in Plant MicroRNA Precursors
Source: Biomed Res Int. 2017 Feb 9;2017:6783010. doi: 10.1155/2017/6783010 (PMC5322449; doi:10.1155/2017/6783010)
Supplement: Supplementary file 1 — Supplementary Material include information about WebLOGO plot of nucleotide frequencies for second-cut regions (Fig S1), number of particular sequence motifs in the first-cut regions (Fig S2), percentage of each nucleotide on specific position in the vicinity of miRNA:miRNA∗ duplex for first-cut regions (Tab S1 & S2) and for random sequences (Tab S3), and number of particular secondary structure motifs in first- and second-cut regions (Fig S3). [file 6783010.f1.pdf]

Supplementary material

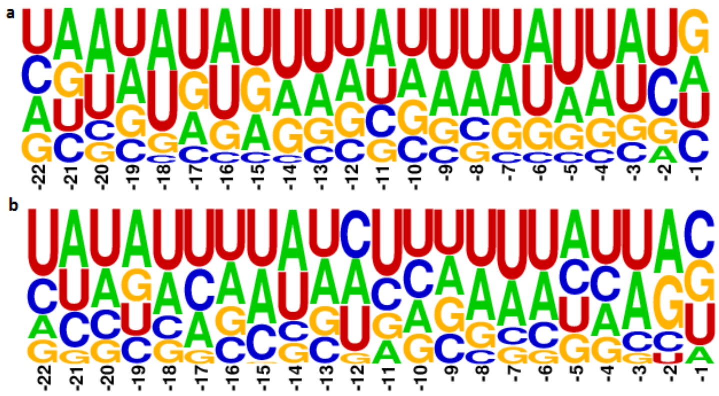

Figure S1. WebLOGO plots of nucleotide frequency in miRNA:miRNA* vicinity on the side of the second cut. (a)regAC, (b)regBD.

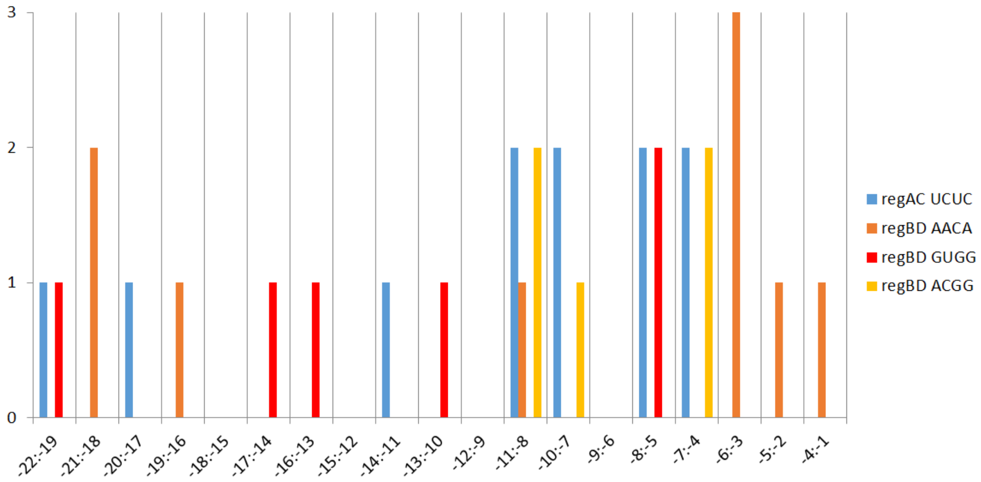

Figure S2. A number of particular sequence motifs in the first-cut regions *regAC* and *regBD*.

Table S1. Percentage of each nucleotide occurrence in the first-cut region *regAC* provided by WebLOGO.

Position	A	C	G	U
**-22**	22	6	36	36
**-21**	28	16	18	38
**-20**	36	18	10	36
**-19**	30	8	22	40
**-18**	36	28	8	28
**-17**	36	6	34	24
**-16**	24	8	44	24
**-15**	26	30	20	24
**-14**	28	16	24	32
**-13**	40	20	26	14
**-12**	20	24	30	26
**-11**	18	16	20	46
**-10**	32	10	28	30
**-9**	14	18	34	34
**-8**	26	22	18	34
**-7**	16	30	26	28
**-6**	28	18	26	28
**-5**	14	26	14	46
**-4**	30	12	26	32
**-3**	20	14	36	30
**-2**	18	12	44	26
**-1**	36	14	24	26

Table S2. Percentage of each nucleotide occurrence in the first-cut region *regBD* provided by WebLOGO.

Position	A	C	G	U
**-22**	18	28	16	38
**-21**	30	12	12	46
**-20**	26	22	16	36
**-19**	28	24	16	32
**-18**	28	22	12	38
**-17**	20	26	20	34
**-16**	10	36	34	20
**-15**	16	24	20	40
**-14**	22	22	18	38
**-13**	20	28	14	38
**-12**	24	18	8	50
**-11**	26	18	26	30
**-10**	16	24	18	42
**-9**	14	30	20	36
**-8**	26	16	28	30
**-7**	26	36	14	24
**-6**	28	22	22	28
**-5**	42	8	28	22
**-4**	30	30	24	16
**-3**	26	38	2	34
**-2**	20	42	14	24
**-1**	26	34	12	28

Table S3. **Percentage of each nucleotide occurrence in random sequences provided by WebLOGO.**

Position	A	C	G	U
**-22**	24	26	26	24
**-21**	34	22	22	22
**-20**	32	20	30	18
**-19**	28	16	28	28
**-18**	26	24	18	32
**-17**	34	20	32	14
**-16**	24	20	32	24
**-15**	38	26	16	20
**-14**	24	38	10	28
**-13**	24	40	20	16
**-12**	22	30	22	26
**-11**	24	22	20	34
**-10**	28	32	26	14
**-9**	24	16	32	28
**-8**	18	28	30	24
**-7**	22	24	32	22
**-6**	20	24	28	28
**-5**	32	26	28	14
**-4**	16	32	26	26
**-3**	20	24	32	24
**-2**	30	28	22	20
**-1**	26	28	22	24

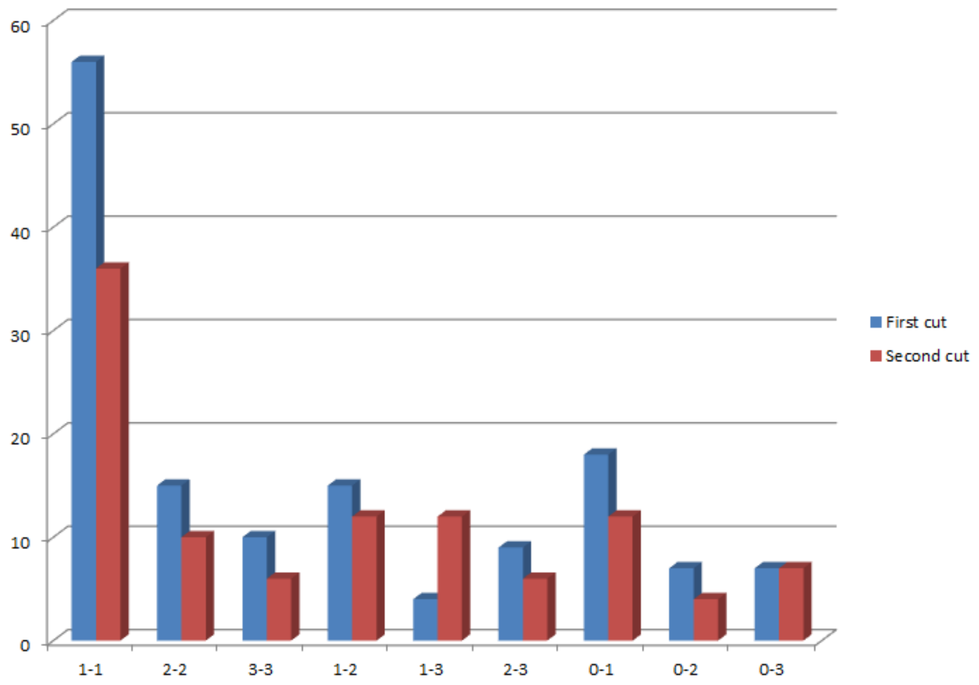

Figure S3. **A number of particular secondary structure motifs (bulges and internal loops) in the first-cut (blue) and the second-cut (red) region.**
